# Schizophrenia and magnetic resonance imaging research: A scientometric analysis during 2014 to 2023

**DOI:** 10.1097/MD.0000000000039710

**Published:** 2024-10-25

**Authors:** Lu Jin, Yuchao Jiang, Hongxing Hu, Yunling Wang, Songnian Fu, Bin Xu, Xiyao Sun, Shuaishuai Gao, Hongmei Wang, Cong Zhao, Ruixue Yang, Wei Zhao, Qizhong Yi

**Affiliations:** a Psychological Medicine Center, The First Affiliated Hospital of Xinjiang Medical University, Urumqi, China; b Xinjiang Clinical Research Center for Mental Health, Urumqi, China; c Institute of Science and Technology for Brain Inspired Intelligence, Fudan University, Shanghai, China; d Department of Magnetic Resonance Imaging, Center of Imaging, The First Affiliated Hospital of Xinjiang Medical University, Urumqi, China.; e Guang Dong Peizheng College, Guang Dong, China; f Xinjiang Medical University, Urumqi, China

**Keywords:** bibliometrics, CiteSpace, magnetic resonance imaging, schizophrenia, VOSviewer

## Abstract

**Background:**

Recently, magnetic resonance imaging (MRI) has emerged as a leading technique for investigating schizophrenia (SZ) pathological mechanisms, prompting an increase in related studies. This study aims to examine the field’s research status and trends via bibliometric analysis.

**Method:**

The publications on SZ and MRI over the past decade were retrieved from the Web of Science Core Collection (WOSCC) On October 15, 2023. CiteSpace and VOSviewer were used to conduct scientometric and visualized analysis, covering countries, institutions, authors, journals, co-cited literature, and keywords.

**Results:**

A total of 4840 publications were retrieved from 2014 to 2023. The United States leads with 1863 articles, followed by China with 1127 articles. King’s College London had the highest number of publications, with 332 articles. Schizophrenia Research ranks first in the journal that published the research on schizophrenia and MRI, the most published journal, Neuroimage is the most cited journal. Calhoun is the most prolific author with 145 articles, and Fischl is the most cited author, receiving 1188 citations. The literature co-citation network (2014 to 2023) revealed 16 clusters with robust structure (*Q* = 0.8719) and high confidence (*S* = 0.9421) involving MRI studies of SZ, genetic imaging and treatment of schizophrenia. Keywords include MRI, psychosis and functional magnetic resonance imaging (fMRI), MRI and neuroimaging, MRI and neuroimaging and white matter and diffusion tensor imaging.

**Conclusion:**

This study offers an overview of the research status and trends of publications on SZ and MRI, aiming to inspire future research directions.

## 1. Introduction

Schizophrenia (SZ) is a severe mental disorder characterized by multidimensional psychotic syndromes, including both positive and negative symptoms (i.e., delusions, hallucinations, and disordered behavior), as well as emotional and cognitive impairments,^[1]^ with a lifetime risk of about 1%, and is the leading cause of disability globally, affecting 26 million people.^[2]^ Patients with SCZ tend to have significantly lower life expectancy compared to the general population.^[3]^

In recent years, MRI has become a key technique for investigating the pathological mechanisms of schizophrenia. Substantial evidence has accrued for widespread functional and structural brain abnormalities in SZ. Structural magnetic resonance imaging (sMRI) studies have revealed that widespread gray matter atrophy in patients with schizophrenia, affecting subcortical regions like the thalamus and basal ganglia, cortical areas including the frontal, temporal, and occipital lobes, and the cerebellum.^[4–7]^ Diffusion tensor imaging studies have shown extensive white matter microstructure damage in SZ patients,^[8,9]^ associated with impaired cognitive function, psychiatric symptoms, and genetic factors.^[10,11]^ Functional MRI studies also indicated aberrant functional connectivity in core large-scale neurocognitive networks.^[12–14]^ With the continuous accumulation of relevant literature, conducting a bibliometric analysis to systematically review and summarize research trends, evolution, frontiers, and hotspots is crucial, providing valuable insights for future research.

Bibliometrics involves the visual analysis of extensive literature data within a specific research field using knowledge graphs. Unlike traditional literature reviews, bibliometrics more intuitively displays the evolution and structural relationships of hot topics in a research field. VOSviewer and CiteSpace are key tools in bibliometric analysis. In this study, the Web of Science database served as the literature source, and bibliometric methods was used to analyze the related research on MRI and schizophrenia in the past decade, so as to summarize the current research status, hot spots and the evolution trend of hot spots.

## 2. Methods

### 2.1. Data acquisition and search strategy

**Figure 1.**
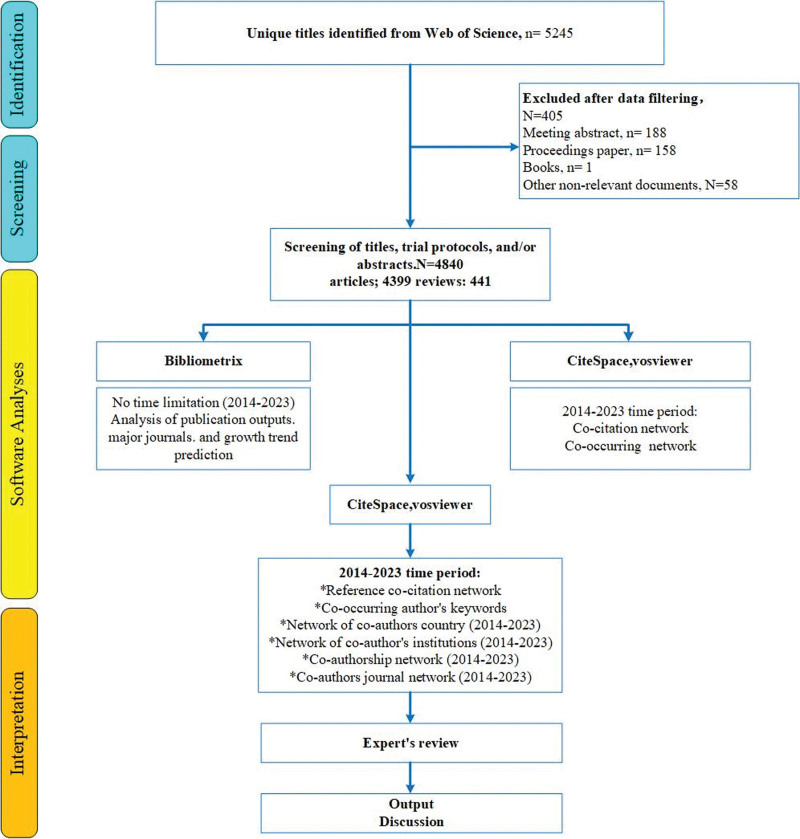
Flow diagram of the bibliometric analysis.

The literature search for this study was conducted using the Web of Science Core Collection (WOSCC) database, a highly preferred source for scientometric analysis due to its comprehensive references and citations.^[15]^ The search strategy was as follows: Topic:((TS=(“magnetic resonance imaging”)) OR TS = (MRI)) AND TS = (Schizophrenia) AND Document Type: (Article OR Review Article) AND Publication Years = 2014-2023. The past decade was chosen as the study’s research period to allow for the identification of the latest research trends through analysis of recent publications. A total of 4840 publications were included and analyzed using scientometric method. The search strategy is depicted in Figure 1. Ethical approval was not required for this bibliometric analysis because it did not involve direct human or animal subjects; it relied solely on public data and literature.

### 2.2. Data analysis

All retrieved data were exported into TXT format for further visual analysis using bibliometric software, such as CiteSpaceV (6.2.R2) and VOSviewer (1.6.19). VOSviewer is a tool for creating and viewing bibliometric maps, capable of generating author or journal maps from citation data, and keyword maps from co-occurrence data.^[16]^ VOSviewer was used in this study to analyze the number of national publications, cooperation conditions and visual literature analysis, including co-occurrence analysis and keyword co-occurrence analysis among countries and institutions. Co-authorship analysis reveals collaboration levels and connection strengths between elements, indicating key countries, institutions, or authors in a study. Keyword co-occurrence analysis can determine the relationship among keywords by calculating the occurrence times of certain keywords in different literatures, clarify the direction and trend of a certain research, and predict the future development trend of a certain research. CiteSpace software can perform co-reference network analysis and visualization of the results, generate co-clustering maps, and perform frequency statistics for co-occurrence keywords and other items. In this study, it facilitated keyword clustering, keyword timeline graph and keyword emergence analysis.

## 3. Results

### 3.1. Annual publications

**Figure 2.**
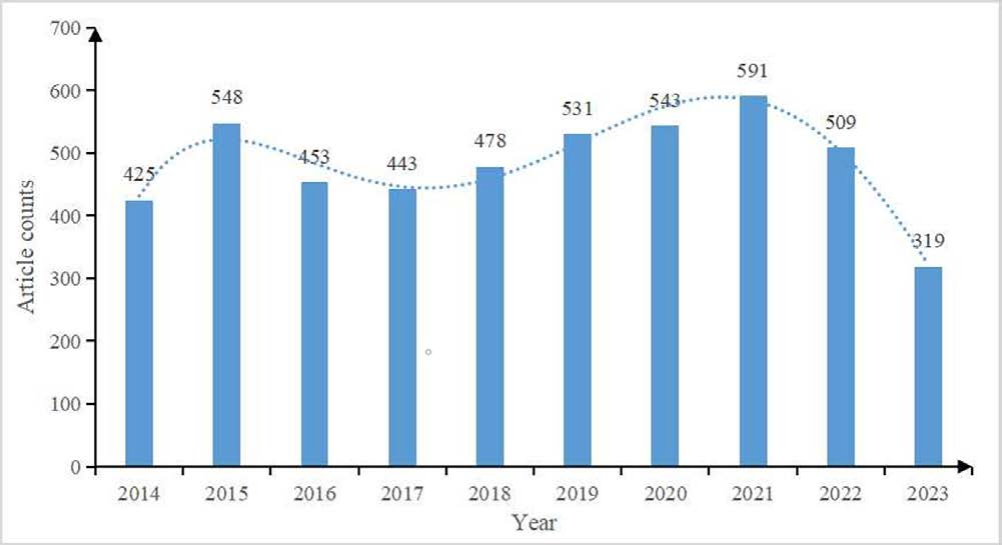
Number of annual publications on SZ and MRI by years.

From 2014 to 2023, a total of 4840 publications meeting the search criteria were included for further analysis. The annual publication is shown in Figure 2. Although the annual publication volume of schizophrenia and MRI fluctuated, there was an overall upward trend.

### 3.2. Country publications and co-operation analysis

**Figure 3.**
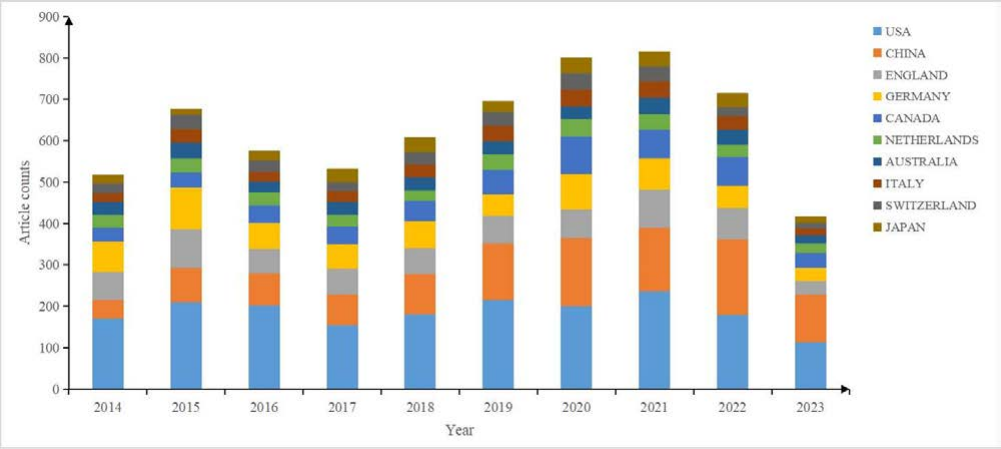
Annual distribution of publications on SZ and MRI among the top 10 countries.

**Figure 4.**
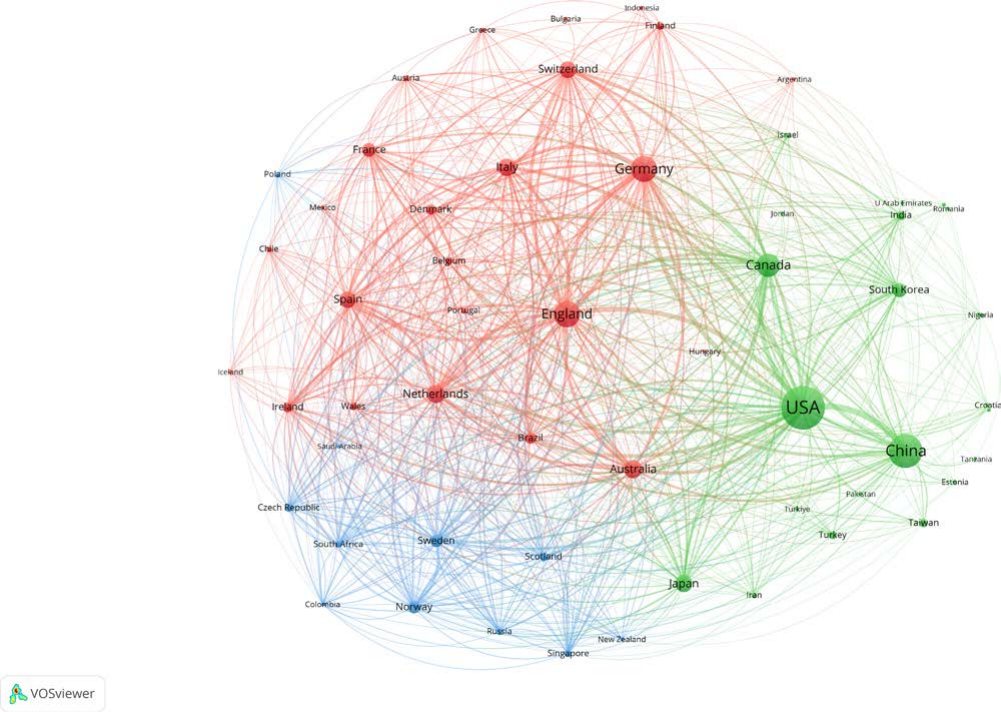
Cooperation network of countries and regions contributed to publications on SZ and MRI.

Among the top 10 countries, the United States consistently held the largest number of publications, while the number of publications in China increasing annually over the past decade (Fig. 3). Table 1 shows that the leading country or regions in terms of the number of publications is the United States (1863, 38.5%), followed by China (1127, 23.2%), England (683, 14.1%), Germany (657, 13.6%), and Canada (530, 11.0%). The United States recorded the highest total citations (52,384), followed by the England (21,192) and Germany (21,102). In the cooperation network of countries and regions (Fig. 4), node size represents the number of publications, and link strength signifies the closeness of collaboration. Countries and regions with the top 5 link strength were the United States, England, Germany, Canada, the Netherlands, indicating close cooperation among them.

**Table 1:** The top 10 countries and regions contributing to the publications on SZ and MRI.

Rank	Country/regions	Publications counts	Citations	Avg. citations	Total link strength
1	United States	1863	52,384	28.12	2362
2	China	1127	19,652	17.44	956
3	England	683	21,192	31.03	1726
4	Germany	657	21,102	32.12	1490
5	Canada	530	13,409	25.30	1157
6	Netherlands	322	10,415	32.34	1052
7	Australia	313	10,436	33.34	893
8	Italy	304	8773	28.86	912
9	Japan	281	5258	18.71	454
10	Switzerland	280	8704	31.09	826

### 3.3. Institution analysis

**Figure 5.**
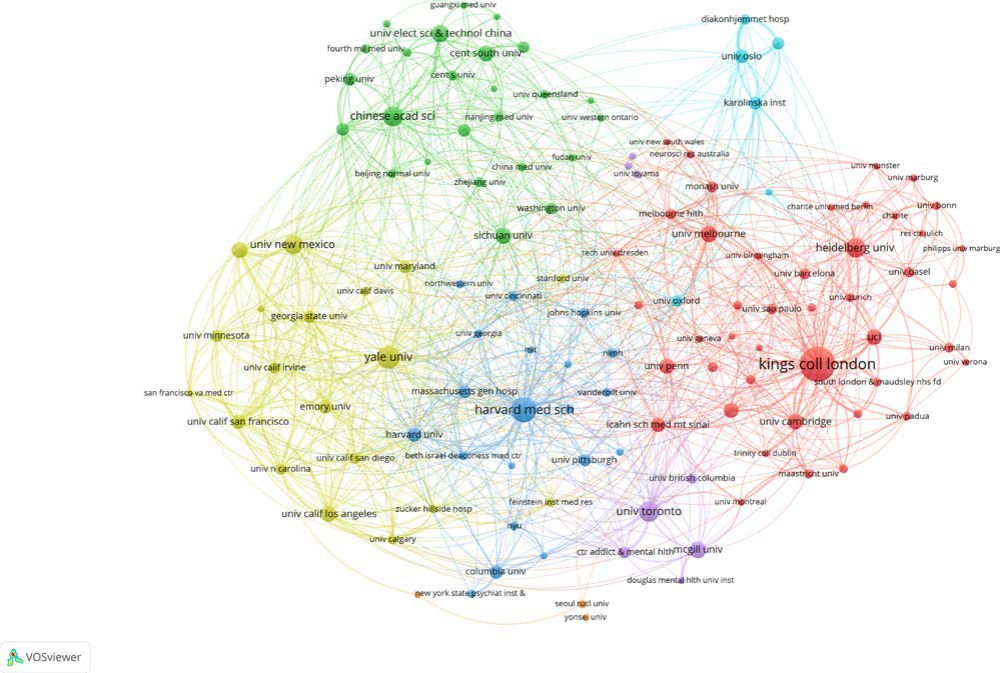
Co-analysis network map of institutions in the field of SZ and MRI.

As shown in Table 2, King’s College London had the largest number of publications (332, 6.86%), followed by Harvard Medical School (210, 4.34%), Yale University (179, 3.7%), University of Toronto (151, 3.12%), and Chinese Acad Sci (148, 3.06%). King’s College London is the most cited institution, with 9544 citations, followed by Yale University (7454) and The University of New Mexico (6355). Figure 5 shows that the institutions with the top 5 link strength are Yale University, King’s College London, Harvard Medical School, The University of New Mexico and Chinese Acad Sci. There is frequent cooperation between these institutions.

**Table 2:** The top 10 institutions contributing to publications on SZ and MRI.

Rank	Institutions	Publications counts	Citations	Avg. citations	Total link strength
1	King’s College London	332	9544	28.747	654
2	Harvard Medical School	210	3218	15.3238	578
3	Yale University	179	7454	41.6425	686
4	University of Toronto	151	3260	21.5894	339
5	Chinese Acad Sci	148	3308	22.3514	359
6	Heidelberg University	143	4256	29.7622	228
7	University of New Mexico	139	6355	45.7194	440
8	University of Electronic Science and Technology of China	117	2393	20.453	197
9	McGill University	113	2465	21.8142	201
10	University of Melbourne	110	2993	27.2091	311

### 3.4. Journals and cited journals Analysis

**Figure 6.**
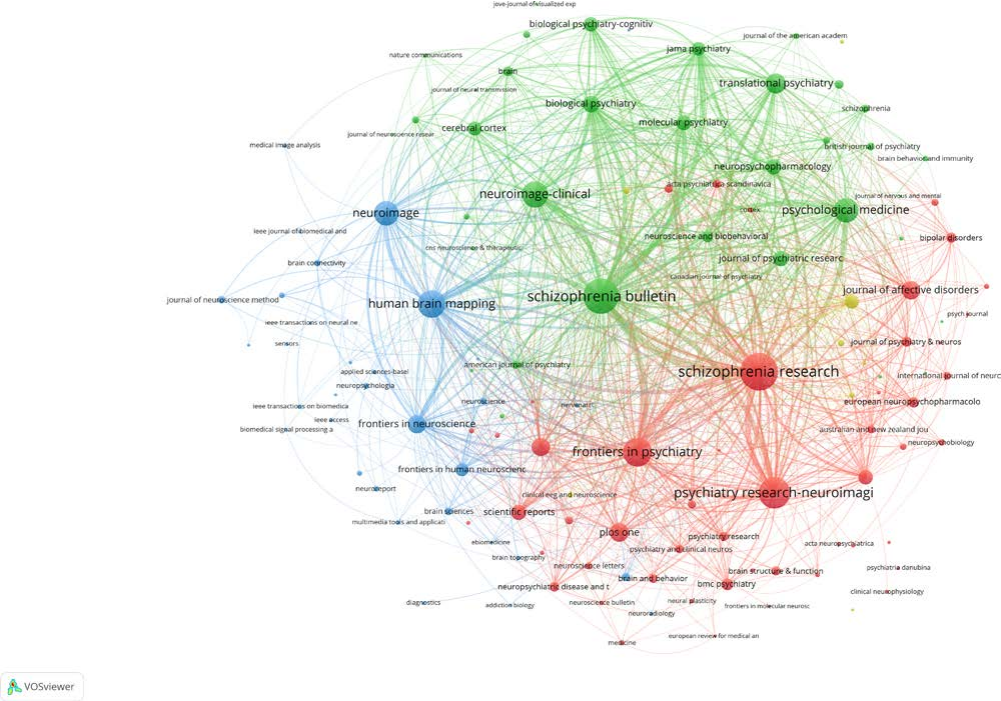
Journals analysis in the field of SZ and MRI.

The publications on SZ and MRI research were published across 589 journals, with 132 journals publishing more than 5 papers each (Fig. 6). Co-citation analysis of journals intends to identify journals with greater influence. Table 3 displays the top 10 cited journals and their most recent IF. The most cited journals was Neuroimage with 4256 times, and the IF was 7, followed by Biological Psychiatry (3710 times), Schizophrenia Research (3661 times), Schizophrenia Bulletin (3418 times) and American Journal of Psychiatry (3417 times). Regarding Journal Citation Rankings (JCR), 8 journals are classified as Q1, and 2 journals belong to Q2. Concerning publication location, 9 of the top 10 journals are based in the United States, with 1 in the Netherlands.

**Table 3:** The top 10 co-cited journals on SZ and MRI from 2014 to 2023.

RANK	Journal title	Total number of citations	Centrality	Impact factor (2022)	Quartile in category (2022)
1	Neuroimage	4256	0.34	7	Q1
2	Biological Psychiatry	3710	0.09	11.9	Q1
3	Schizophrenia Research	3661	0.12	4.5	Q2
4	Schizophrenia Bulletin	3418	0.1	8.1	Q1
5	American Journal of Psychiatry	3417	0.07	17.8	Q1
6	Human Brain Mapping	2900	0.02	5.4	Q1
7	Archives of General Psychiatry	2849	0.04	15.56	Q1
8	Proceedings of The National Academy of Sciences of The United States of America	2600	0.02	12	Q1
9	Plos One	2493	0.01	3.8	Q2
10	Journal of Neuroscience	2486	0.01	6.2	Q1

### 3.5. Author co-operation and co-citation analysis

**Figure 7.**
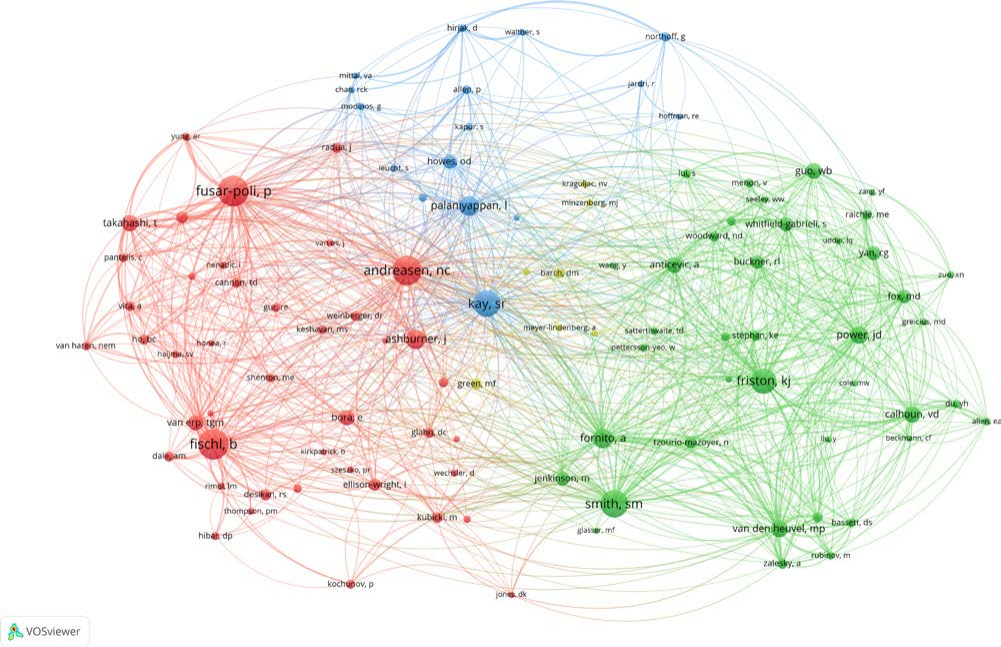
Cooperation network of cited authors contributed to publications on SZ and MRI.

The authors’ cooperation and co-citation analysis aims to identify researchers who have a greater impact in the research field. Table 4 enumerates the top 10 productive authors and co-cited authors. Among the 4840 papers, the top 10 authors published 612 papers, accounting for 12.6% of the total number of publications. Since 2014, Calhoun has published 145 papers and ranked first in the list, followed by Guo (57 papers) and Zhao (55 papers). Fischl is the most cited author with 1188 citations, followed by Fusar-poli (1172 citations) and Andreasen (1128 citations). Figure 7 displays a total of 108 authors cited more than 200 times each.

**Table 4:** The top 10 productive authors and co-cited authors on SZ and MRI studies from 2014 to 2023.

Rank	Author	Publication counts	Co-cited author	Total number of citations
1	Calhoun, Vince D	145	Fischl B	1188
2	Guo, Wenbin	57	Fusar-poli P	1172
3	Zhao, Jingping	55	Andreasen NC	1128
4	Andreassen, Ole A	54	Smith SM	1053
5	Mathalon, Daniel H.	53	Kay SR	1023
6	Agartz, Ingrid	52	Friston KJ	974
7	Liu, Feng	52	Fornito A	732
8	Pantelis, Christos	49	Palaniyappan L	729
9	Mcguire, Philip	48	Ashburner J	712
10	Gong, Qiyong	47	Power JD	638

### 3.6. Co-cited reference analysis

#### 3.6.1. Most cited papers

Citation analysis is a crucial method for evaluating highly cited articles, showcasing their impact in specific research fields. Table 5 presents the top 10 co-cited references. Kay et al’s article in Schizophrenia Bulletin had the maximum number of citations (938 citations), followed by Desikan’s article in Neuroimage (382 citations), and Tzourio-Mazoyer et al’s article in Neuroimage (372 citations).

**Table 5:** The top 10 co-cited references.

Rank	Title	First author	Journal	Year	Citations
1	The Positive and Negative Syndrome Scale for Schizophrenia	Kay SR	Schizophrenia Bulletin	1987	938
2	An automated labeling system for subdividing the human cerebral cortex on MRI scans into gyral based regions of interest	Desikan	Neuroimage	2006	382
3	Automated anatomical labeling of activations in SPM using a macroscopic anatomical parcellation of the MNI MRI single-subject brain	Tzourio-Mazoyer	Neuroimage	2002	372
4	Spurious but systematic correlations in functional connectivity MRI networks arise from subject motion	Power	Neuroimage	2012	352
5	Cortical surface-based analysis - I. Segmentation and surface reconstruction	Dale	Neuroimage	1999	302
6	Whole brain segmentation: Automated labeling of neuroanatomical structures in the human brain	Fischl	Neuron	2002	298
7	The assessment and analysis of handedness: The Edinburgh inventory	Oldfield	Neuropsychologia	1971	279
8	A fast diffeomorphic image registration algorithm	Ashburner	Neuroimage	2007	269
9	Brain Volumes in Schizophrenia: A Meta-Analysis in Over 18 000 Subjects	Haijma	Schizophrenia Bulletin	2013	263
10	Hyperactivity and hyperconnectivity of the default network in schizophrenia and in first-degree relatives of persons with schizophrenia	Whitfield-Gabrieli	Proceedings of The National Academy of Sciences of The United States of America	2009	247

#### 3.6.2. Co-cited reference cluster analysis

**Figure 8.**
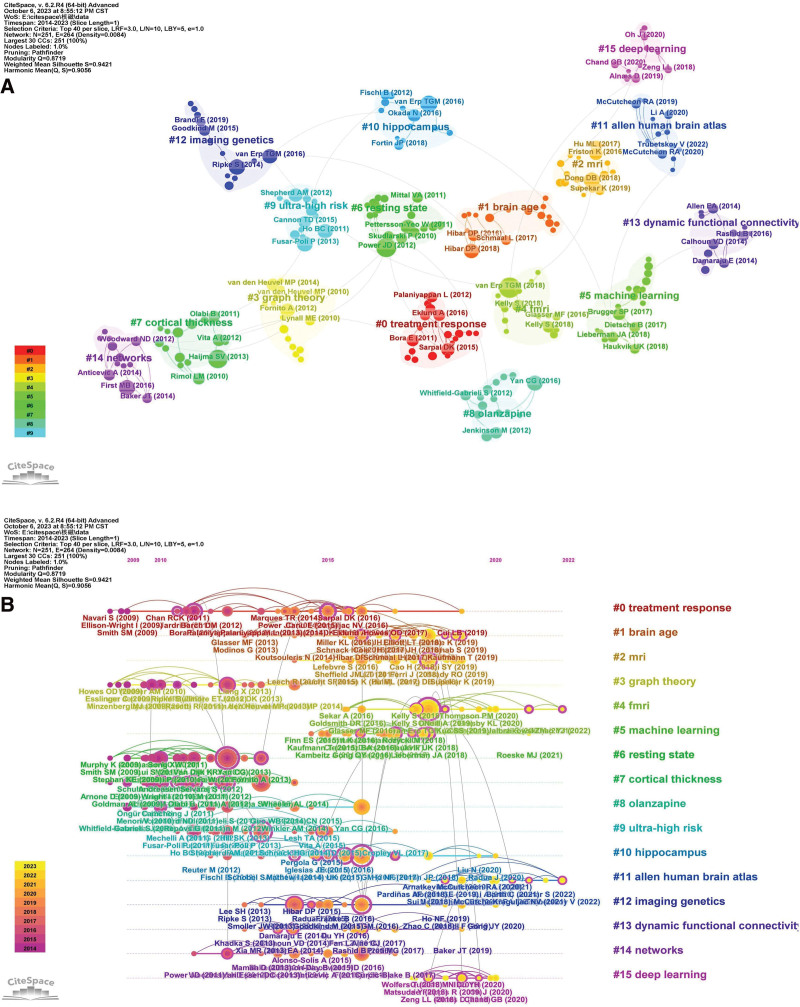
(A) Clustered network of co-cited references of the publications on SZ and MRI. (B) Timeline visualization of clustered network of co-citation references.

Co-cited references cluster analysis can segment the literature into clusters representing different subject concentrations. CtieSpace was utilized to analyze references and their co-citation network and clustering map (Fig. 8A). The map shows 16 different clusters, and the *Q* and *S* values (*Q* = 0.8719; *S* value = 0.9421) are significant, indicating that these clusters have high confidence. The 5 largest clusters include treatment response, brain age, MRI, graph theory, and fMRI.

Three major research trends were found. The first trend encompassed MRI studies of SZ, including #2 “Mri,” #3 “graph theory,” #4 “fMRI,” #5 “machine learning,” #6 “resting state,” #7 “cortical thickness,” #10 “hippocampus,” #13 “dynamic functional connectivity,” #14 “networks,” #15 “deep learning.” The research methods are divided into traditional methods (#3 “graph theory”) and machine learning analysis methods (#5 “Machine learning,” #15 “deep learning”). The second trend involved genetic imaging of schizophrenia, including #9 “ultra-high risk,” #11 “allen human brain atlas,” and #12 “imaging genetics.” The third trend was related to the treatment of schizophrenia, including #0 “treatment response” and #8 “olanzapine.”

The timeline view displays the distribution and evolution of nodes in each cluster over time, highlighting shifts in research focus and uncovering the latest trends. The timeline visualization of the cluster analysis of co-citation references is shown in Figure 8B. #4 fMRI, #6 resting state, and #10 hippocampus are the largest clusters. #5 “Machine learning” and #12 “imaging genetics” are the hot spots of current research.

### 3.7. Keyword analysis

**Figure 9.**
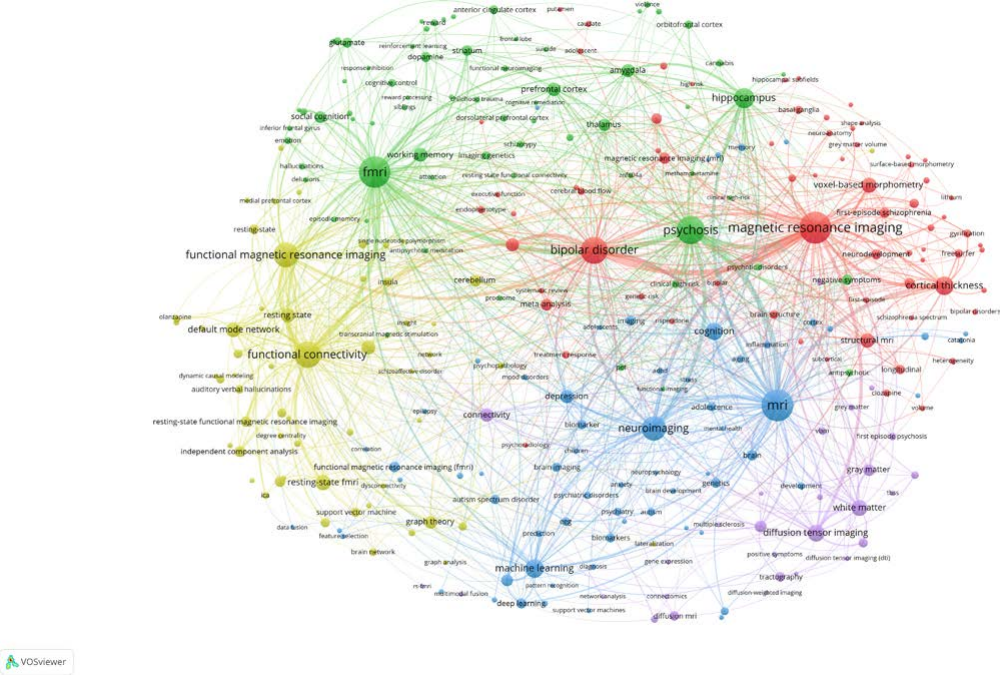
The keyword co-occurrence map.

Keyword analysis is used to mine core keywords, identify co-occurrence links, classify keywords into clusters, and understand the research hotspots in the target field. The network of keyword co-occurrence cluster analysis is displayed in Figure 9. A total of 6739 keywords were extracted from all literatures. Around 288 keywords with frequencies over 10 were analyzed. As shown in the figure, the largest node in the co-occurrence diagram is MRI, which is consistent with the theme of this study. The keywords can be divided into 5 clusters: Cluster 1 is colored in red, with the main keywords focusing on MRI such as “bipolar disorder,” “cortical thickness,” “structural MRI,” “voxel-based morphometry.” Cluster 2 in green focused on psychosis and fMRI, with the main keywords “hippocampus,” “thalamus,” “prefronatal cortex,” “amygala.” Cluster 3 in blue color focused on MRI and neuroimaging. Cluster 4 in yellow color focused on the functional MRI and functional connectivity. Cluster 5 in purple color focused on white matter and diffusion tensor imaging.

**Figure 10.**
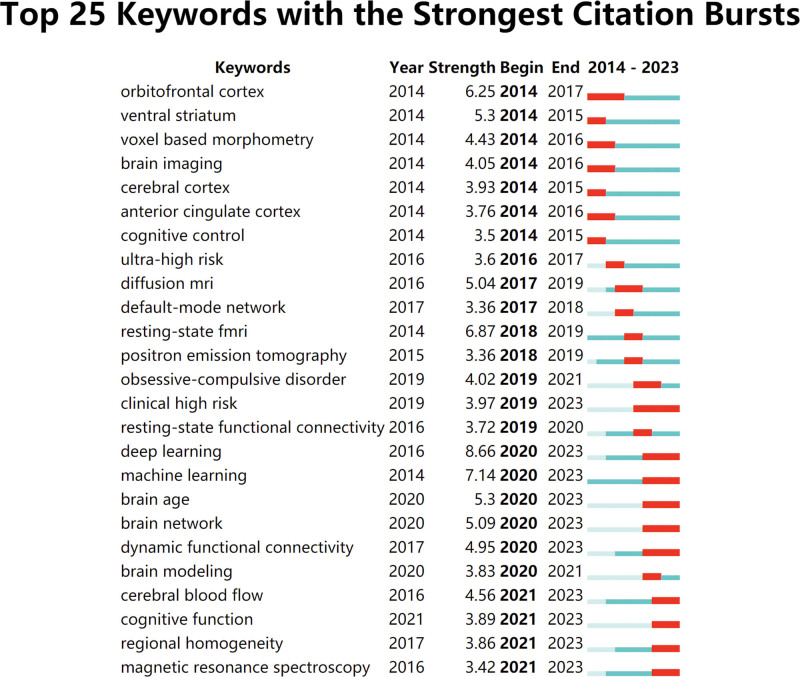
visualization map of top 25 keywords with the strongest citations bursts.

Figure 10 shows the top 25 keywords with the strongest citation bursts from 2014 to 2023. As shown in the figure, deep learning has the maximum burst strength, followed by machine learning, resting-state fMRI, orbitofrontal cortex, ventral striatum.

## 4. Discussion

### 4.1. General information

This study conducted a bibliometric analysis of the literature from 2014 to 2023 on patients with schizophrenia and MRI, providing an overview of the study. Our findings indicate that interest in the target field has remained strong, with an overall upward trend. The USA, leading the top 10 countries/regions in research contributions, accounted for 38.5% of the total papers, followed by China and England. The close cooperation of the United States, England, Germany, Canada and the Netherlands shows that they have made a significant contribution to research in this field. Although China ranks second in SZ and MRI publications, its collaborative intensity is relatively low, suggesting a need for stronger international cooperation. In terms of institutional contributions, King’s College London, Harvard Medical School, and Yale University led the rankings.King’s College London was the most cited institution, with Yale University and The University of New Mexico following.The top 3 journals in terms of publications were Schizophrenia Research, Schizophrenia Bulletin and Psychiatry Research-neuroimaging. And the top 3 cited journals were Neuroimage, Biological Psychiatry and Schizophrenia Research. Calhoun was identified as the most prolific scholar, having published the highest number of articles. Calhoun was identified as the most prolific scholar, having published the highest number of articles. Visualization of the most active research institutions and teams aids in identifying potential collaborators.

### 4.2. Main research content of publications

To reveal the latest research trends, we obtained literature co-citation networks from 2014 to 2023. Through co-cited literature analysis, CiteSpace identified 16 clusters, which fall into 3 main research trends: MRI studies of schizophrenia, genetic imaging of schizophrenia, and schizophrenia treatment. The first major study trend involved the MRI studies of SZ, with different structural imaging techniques including sMRI, diffusion magnetic resonance imaging (dMRI), and functional magnetic resonance imaging (fMRI), to investigate and explore the structure and function of the brain from different angles. The initial research on SZ classification mainly used single-mode image data to classify diseases, yet relying solely on single-mode data proves insufficient for comprehensive brain disease research and analysis. Following neuroimaging technology advancements, researchers have shifted focus to integrating multi-modal image data. This approach combines structural and functional MRI to enhance complementarity and employs machine learning with multi-modal MRI for more accurate mental disease diagnosis and classification. Zhuang et al combined sMRI, diffusion tensor imaging and resting fMRI data to distinguish untreated schizophrenia patients from normal subjects, who used sparse coding for feature selection and multi-core SVM for feature combination and classification, achieving a classification accuracy of 84.29%.^[17]^ Cui et al employed a deep neural network to automatically distinguish schizophrenia patients from healthy individuals based on subjects’ gray matter, white matter and cerebrospinal fluid volumes as classification features, and conducted experiments in 8 independent research centers, with a classification accuracy of 77.19% to 85.74% and a sensitivity of 75.31% to 89.29%.^[18]^ Combining deep learning technology with MRI to assist in the diagnosis and prediction of mental diseases is a current research hotspot. In imaging genetics, researchers utilize genetic information and MRI data to investigate how schizophrenia risk gene genotypes modulate brain structure and function, thereby elucidating the biological mechanisms of these genes. A high polygenic risk score for schizophrenia is associated with a thinner ventromedial prefrontal cortex (vmPFC), suggesting that genetic factors may increase the risk of schizophrenia by affecting the integrity of the vmPFC.^[19]^ Shen et al studied the regulatory effects of apolipoprotein gene and sorting protein-associated receptor L1 gene on the functional connectivity of hippocampus in healthy young people based on 323 healthy young subjects.^[20]^ In treatment of schizophrenia, the main research is to study the effects of antipsychotic treatment on brain structure and function. McNabb et al found that compared with healthy controls and patients responding to treatment, TRS patients had increased functional connectivity in some regions of the resting sensorimotor network, especially in the striatum, which meant that the treatment response was worsened.^[21–23]^

Burst detection analysis is a crucial method for tracking the evolution of research hotspots within academic fields of interest. Among the top 25 keywords with the strongest citation bursts from 2014 to 2023, deep learning has the maximum burst strength. Deep learning technology combined with MRI to assist the diagnosis and prediction of mental diseases is 1 of the current research focus. Deep learning is a tool of machine learning which are capable of characterizing discriminant patterns and automatically learning the best representation from neuroimaging data. 1 of the most commonly used input features of deep learning is functional network connections based on brain atlas or ICA computing.^[24]^ Pinaya et al extracted the cortical thickness and anatomical volume of structural images of people with schizophrenia and normal people, and completed the classification of patients with schizophrenia using a deep neural network DBN DN-DNN, with an accuracy of 73.6%.^[25]^ Kim et al used deep neural networks to classify schizophrenia patients based on resting-state MRI data and found abnormal in functional connectivity in patients with schizophrenia.^[26]^

### 4.3. Strengths and limitations

The study analyze the publications about SZ and MRI during 2014 to 2023 using bibliometrics analysis, clarifying research trends and hotspots with a view to providing useful references for future related research. It should be noted that Duan et al conducted a bibliometric analysis about MRI studies of SCZ from 2004 to 2018 and divided the publications (3772 studies) retrieved from PubMed into 3 periods (2004 to 2008, 2009 to 2013, and 2014 to 2018) and analyzed the major Medical Subject Headings (MeSH) terms/MeSH subheadings.^[27]^ Our study searched the latest literature (4840 publications) in the last ten years from WOSCC database for scientometric analysis, covering countries, institutions, authors, journals, co-cited reference analysis and keyword analysis offers an overview of the research status and identify the latest research trends on SZ and MRI.

However, the study has several limitations. Firstly, it excluded literature in languages other than English, potentially underestimating contributions from non-English-speaking countries. Secondly, the data were solely sourced from the WOSCC, leading to potential incompleteness and overlooking literature in other databases. With the continuous development of visualization software, multi-database processing is expected to be realized, and the subsequent research can be further improved on this basis.

## 5. Conclusion

This study conduct the bibliometric analysis of publications SZ and MRI publications over the past decade performed by VOSviewer and CiteSpace software. The findings identify the major countries, institutions, journals, authors, etc, that are driving progress in this field. The research primarily focused on MRI studies of SZ, genetic imaging of schizophrenia and treatment of schizophrenia. Emerging trends focus on the application of new techniques such as deep learning in neuroimaging studies. This study encapsulates the current status and trends of SZ and MRI research, offering insights and directions for future studies.

## Acknowledgments

The present study was sponsored by Natural Science Foundation of Xinjiang Uygur Autonomous Region (Grant No. 2022D01D64 and Grant No. 2022D01C749) and Science and Technology Support Projects in Xinjiang Uygur Autonomous Region (Grant No. 2020E0275) and Science and Technology Assistance Project for Xinjiang Uygur Autonomous Region (Grant No. 2022E02055) and Xinjiang Medical University First Affiliated Hospital Youth Research Launch Special Fund (Grant No. 2022YFY-QKQN-71).

## Author contributions

**Conceptualization:** Bin Xu.

**Data curation:** Xiyao Sun, Shuaishuai Gao, Ruixue Yang.

**Formal analysis:** Hongmei Wang.

**Investigation:** Yunling Wang.

**Methodology:** Lu Jin.

**Project administration:** Qizhong Yi.

**Resources:** Songnian Fu.

**Software:** Yuchao Jiang.

**Validation:** Cong Zhao, Wei Zhao.

**Visualization:** Hongxing Hu.

**Writing – original draft:** Lu Jin.

**Writing – review & editing:** Hongxing Hu, Songnian Fu, Qizhong Yi.
